# Tailoring the Microstructure and Properties of HiPIMS-Deposited DLC-Cr Nanocomposite Films via Chromium Doping

**DOI:** 10.3390/nano16020150

**Published:** 2026-01-22

**Authors:** Jicheng Ding, Wenjian Zhuang, Qingye Wang, Qi Wang, Haijuan Mei, Dongcai Zhao, Xingguang Liu, Jun Zheng

**Affiliations:** 1 China International Science and Technology Cooperation Base on Intelligent Equipment Manufacturing in Special Service Environment, Anhui University of Technology, Maanshan 243002, China; 2 Key Laboratory of Green Fabrication and Surface Technology of Advanced Metal Materials, Ministry of Education, Anhui University of Technology, Maanshan 243002, China; zhaodongc@163.com (D.Z.); sdwfcllxg@126.com (X.L.); 3 School of Materials Science and Engineering, Anhui University of Technology, Maanshan 243002, China; zwj23auot@126.com (W.Z.); wangqingye585156@gmail.com (Q.W.); wangqiahut717@163.com (Q.W.); 4 Guangdong Provincial Key Laboratory of Electronic Functional Materials and Devices, Huizhou University, Huizhou 516007, China; haijuanmei@hzu.edu.cn

**Keywords:** DLC film, Cr doping, microstructure, mechanical properties, wettability

## Abstract

Chromium-doped diamond-like carbon (DLC-Cr) nanocomposite films were successfully deposited using a high-power impulse magnetron sputtering (HiPIMS) system. The Cr content in the films was controlled by adjusting the Cr target powers. The influence of Cr content on the microstructure, mechanical properties, tribological performance, and wettability of the films was systematically investigated. The results show that the Cr content and deposition rate of the films increased with increases in the target power. The surface topography of the films evolved from smooth to rough as the Cr target increased from 10 W to 70 W. At low Cr doping rates, the film mainly exhibited an amorphous structure, whereas the nanocomposite structure was formed at proper Cr doping rates. Raman and XPS analyses revealed that Cr incorporation altered the I_D_/I_G_ ratio and promoted the formation of Cr-C bonds, leading to a more graphitic and nanocomposite-like structure. The nanoindentation results show that an optimal Cr content enhances both hardness and elastic modulus, while higher Cr concentrations lead to a decline in mechanical strength due to more graphitization and decreasing stress. Tribological tests exhibited a significant reduction in the friction coefficient (0.21) and wear rate (0.63 × 10^−14^ m^3^/N·m) at a moderate Cr level. Additionally, the surface wettability evolved toward enhanced hydrophilicity with increasing Cr power, as evidenced by reduced water contact angles and increased surface energy. These findings demonstrate that controlled Cr incorporation effectively tailors the structure, stress state, and surface chemistry of DLC films, offering a tunable pathway to achieving optimal mechanical performance and tribological stability for advanced engineering applications.

## 1. Introduction

Diamond-like carbon (DLC) films have attracted extensive research interest over the past few decades owing to their unique combination of mechanical and tribological properties, such as high hardness, a low friction coefficient, superior wear resistance, and excellent chemical inertness [1,2,3,4]. Owing to these properties, DLC films have been applied extensively in various fields, such as cutting and forming tools, biomedical implants, automotive engine components, and microelectromechanical systems (MEMSs) [5,6,7,8]. Despite these advantages, the large-scale industrial adoption of pure DLC films remains restricted by several inherent drawbacks. Specifically, high intrinsic stress often leads to film delamination, poor adhesion to metallic substrates limits durability, and insufficient thermal stability results in performance degradation under elevated temperature conditions [9,10]. These limitations significantly hinder the practical reliability of DLC films in demanding service environments.

To address these issues, extensive efforts have been devoted to tailoring the structure and properties of DLC films. Various strategies have been developed, including multilayer architectures, gradient interfaces, hydrogenation, and element doping [11,12,13,14,15]. Among them, element doping has proven to be particularly effective in tuning the microstructure and mechanical performance of DLC films. Both metallic and non-metallic dopants have been widely explored. Non-metallic dopants such as nitrogen, silicon, and boron can regulate the sp^2^/sp^3^ bonding ratio and mechanical properties, and can improve the thermal stability of DLC films [16,17,18]. For example, in a work by Ramirez et al. [16], the authors deposited DLC films with various N contents by controlling the N_2_ ratio. Their results show that the sp^2^/sp^3^ ratio firstly decreased from 0.9 to 0.3 with an increase in the N_2_ ratio, then increased to 0.8 at a N_2_ ratio of 60%. Meanwhile, the mechanical properties including hardness and elastic modulus were linearly decreased from 37.1 GPa and 337.9 GPa without N_2_ to 16.3 GPa and 135.9 GPa, respectively, at a N_2_ ratio of 60%. This tendency was attributed to changes in film ductility due to the N-doping. In other previous works [17,19], DLC films with tailored Si concentrations were produced under various deposition conditions. The results demonstrate that the incorporation of an optimal amount of Si significantly enhances the mechanical properties, tribological performance, and high-temperature oxidation resistance of the films, as compared to undoped DLC films.

In contrast, metallic dopants are generally classified into two categories: carbide-forming elements (e.g., Cr [8,20], Ti [4,21], W [22], and Mo [15,23]) and non-carbide-forming elements (e.g., Ag [14,24], Cu [25], and Al [26,27]). The former tends to react with carbon to form nanocrystalline carbide phases dispersed within the amorphous carbon matrix, which can relieve residual stress, refine microstructure, and improve hardness and wear resistance. The latter are usually present in a metallic or amorphous state, playing a role in enhancing ductility or promoting self-lubrication. Thus, the selection of dopant species critically determines the structural evolution and resulting properties of DLC-based nanocomposites. Among the diverse metallic dopants, chromium (Cr) has attracted attention due to its moderate atomic size, strong chemical affinity with carbon, and ability to form thermodynamically stable Cr-C bonds. The incorporation of Cr not only modifies the bonding environment of carbon atoms but also promotes the formation of nanocrystalline CrC phases within the amorphous carbon matrix. This dual effect can effectively relieve internal stress, improve adhesion, and enhance the mechanical properties of DLC films. For instance, in the study by Zhou et al. [28], the authors found that Cr content had a significant impact on the structure and friction of Cr/F-DLC film. The introduction of Cr disrupted the carbon matrix structure and the formation of chromium carbide nanocrystals was beneficial for the improvement of the wear resistance of the films. Moreover, the formation of Cr-containing phases has been reported to strengthen the adhesion and enhance the tribological performance of DLC films by promoting the development of stable transfer layers, which effectively reduce friction and consequently improve wear resistance [20]. Beyond the mechanical and tribological benefits, Cr doping may alter surface energy and wettability, further broadening the potential application range of DLC-based films in different service environments. In Wang et al. [29], the authors investigated the hydrophobic behavior of DLC-Cr film on stainless steel bipolar plates and found that all DLC-Cr films had a larger water contact angle than the SUS304 substrate. Meanwhile, the corrosion resistance of DLC-Cr films was also improved and the optimized DLC-Cr film was a good candidate application in polymer electrolyte membrane fuel cells (PEMFCs).

Despite many studies on Cr-doped DLC films, most reported works rely on conventional direct current (DC) or radio frequency (RF) sputtering for carbon deposition, where the ionization degree of carbon species is inherently low. In contrast, the present study employs a hybrid HiPIMS–pulsed DC configuration, in which the carbon target is operated in HiPIMS mode to enhance carbon ionization, while the Cr content is independently regulated by adjusting the Cr target power. Specially, the HiPIMS could offer a high degree of ionization of sputtered species and enhance ion energy control compared to conventional DC sputtering [2,30]. This approach enables a unique growth environment for DLC-Cr films, allowing for systematic investigation of the interplay between carbon structure, carbide formation, stress relaxation, and multifunctional properties, which has not been fully addressed in previous studies.

Accordingly, in this study, Cr-doped DLC (DLC-Cr) nanocomposite films were deposited by a hybrid sputtering system, combining the HiPIMS source and a pulsed direct current (PDC) unit. The microstructure, chemical bonding, mechanical properties, and tribological performance of the DLC-Cr films were systematically investigated. In addition, the wettability behavior of the films was also evaluated by contact angle measurements to understand the surface energy modifications induced by Cr incorporation. The study aims to provide valuable insights into the structure–property relationships in DLC-Cr nanocomposite films and reference for various engineering applications.

## 2. Experimental Details

### 2.1. Films Deposition

The Cr-doped diamond-like carbon (DLC-Cr) films were synthesized on silicon wafer and SUS304 stainless steel substrates using a dual-source deposition platform integrating the high-power impulse magnetron sputtering (HiPIMS) and pulsed DC (PDC) technologies. A high-purity graphite (C) target (99.9%) and a chromium (Cr) target (99.9%) were connected to the HiPIMS generator and PDC power supply, respectively. Before deposition, the substrates were first subjected to sequential ultrasonic cleaning in acetone and ethanol baths (15 min per step), followed by drying with nitrogen gas. Afterward, they were positioned at the chamber center on a rotating substrate holder with a rotation speed of 10 rpm. The base pressure of the chamber was 5 × 10^−3^ Pa and argon was then introduced as the sputtering gas. The working pressure was stabilized at 0.6 Pa via mass flow control, and substrate temperature was maintained at 200 °C. Before depositing the expected films, a two-stage surface pretreatment was necessary. Firstly, the two targets were pre-sputtered to eliminate surface impurities, and then the surfaces of substrates were bombarded with Ar^+^ ions at a DC bias voltage of −700 V to remove native oxides. A thin Cr interlayer was intentionally deposited to enhance the adhesion between the film and the substrate. Subsequently, the average C target power was fixed at 700 W, with HiPIMS parameters set to a 206.6 Hz pulse frequency and a 20% duty cycle. To obtain DLC-Cr films with different Cr contents, the Cr target power was adjusted from 10 to 70 W. All films were prepared with a total deposition time of 180 min, while the substrate bias voltage was kept constant at −100 V.

### 2.2. Film Characterization

The morphology and nanoscale roughness of the films were evaluated through field-emission scanning electron microscopy (FE-SEM, Hitachi S4800, Tokyo, Japan) and atomic force microscopy (Bruker MFM-3D AFM, Santa Barbara, CA, USA), respectively. Chemical composition and bonding configurations were analyzed by X-ray photoelectron spectroscopy (Thermo Scientific ESCALAB 250Xi, Waltham, MA, USA) using monochromatic Al Kα radiation (1486.6 eV) at 15 kV. Survey spectra were collected with a pass energy of 150 eV, while high-resolution spectra were acquired at 20 eV. The binding energy scale was calibrated by setting the adventitious carbon peak to 284.8 eV, with an overall binding energy accuracy of ±0.1 eV. Prior to high-resolution acquisition, the sample surfaces were mildly cleaned using Ar^+^ ion sputtering at 2 keV for 180 s. Fitting of the XPS spectra was performed with XPSPEAK-4.1 software using a sum of the Lorentzian–Gaussian (20:80) function with Shirley background subtraction. The identification of the carbon bonding structure was performed using Raman spectroscopy (XperRam200, Seoul, Republic of Korea) with a 532 nm excitation laser. The obtained Raman spectra were fitted with Gaussian functions after subtracting a linear baseline over the 1000–1800 cm^−1^ range. Cross-sectional microstructure characterization involved focused ion beam sample preparation and high-resolution transmission electron microscopy (FEI Talos F200X, Waltham, MA, USA) operated at 200 kV. Mechanical properties, including hardness (*H*) and elastic modulus (*E*), were quantified via nanoindentation (Bruker Hysitron TI950, Eden Prairie, MN, USA) with a calibrated diamond Berkovich tip (with the area function and frame stiffness calibrated on fused silica). Tests were performed at a 1 mN load with indentation depths restricted to <10% of film thickness to minimize substrate interference, and hardness/modulus were extracted via the Oliver–Pharr method. Ten randomly distributed indentations per sample were averaged for statistical reliability. Residual stresses were derived from substrate curvature measurements using Stoney’s formulation [31]. The tribological behavior of the films was evaluated using a ball-on-disk tribometer with the relative humidity maintained between 40% and 60%. The counterpart was an Al_2_O_3_ ball with a diameter of Φ6 mm and the normal load was 1 N. The sliding tests were conducted at a linear velocity of 8.37 cm/s with a rotation radius of 4 mm. The wear track morphology and cross-section obtained in the tribological tests were observed using a Taylor Hobson^®^ CCI 3D Optical Profiler (Leicester, UK). The wear rate (W) was calculated following the Archard model, expressed as W = V/(N × L), where V denotes the volume loss, N is the normal load, and L corresponds to the total sliding distance. Wettability analysis was conducted via the sessile drop technique (SmartDrop_Plus) with deionized water under ambient conditions.

## 3. Results and Discussion

**Figure 1:**
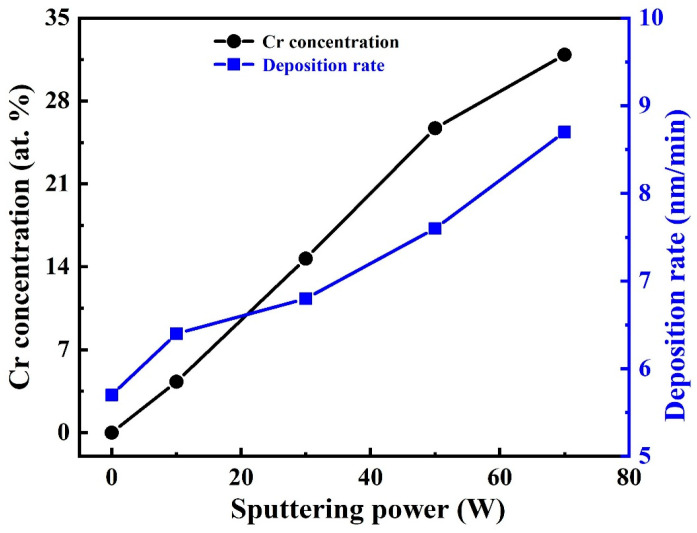
The Cr content and deposition rate of DLC-Cr films with respect to Cr target powers.

Figure 1 illustrates the variations in Cr content and deposition rate of the DLC-Cr films as a function of the Cr target power. As the Cr target power increased from 10 W to 70 W, the Cr content incorporated into the films rose markedly from 4.3 at.% to 31.9 at.%, accompanied by an increase in the deposition rate from 6.4 nm/min to 8.7 nm/min. Consequently, the total film thickness increased gradually from approximately 1.15 μm to 1.57 μm for Cr contents of 4.3 at.%, 14.7 at.%, 25.7 at.%, and 31.9 at.%, respectively. This thickness evolution is consistent with the enhanced deposition rate at higher target powers. These results demonstrate that precise control of the Cr incorporation can be achieved through adjustment of the target power, which is consistent with earlier reports [15,32]. Such behavior is closely related to the characteristics of the power supply discharge. Increasing the target power enhances the average power density applied to the Cr target, which in turn generates a plasma with higher density and consequently leads to the greater flux of sputtered Cr atoms and ions. The intensified flux of Cr species arriving at the substrate per unit time not only contributes to the elevated Cr concentration in the growing film but also results in the enhancement of the overall deposition rate.

**Figure 2:**
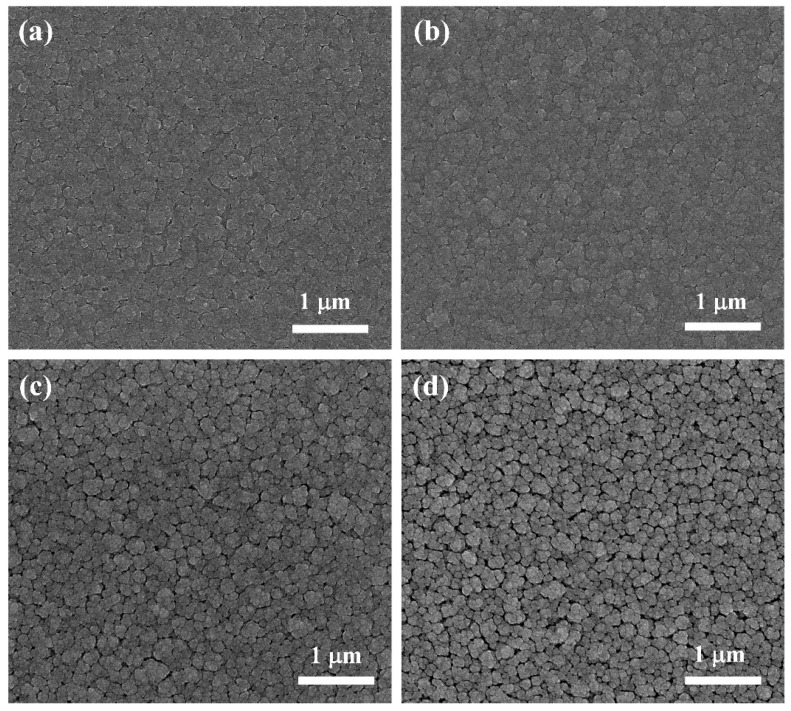
The surface morphologies of DLC-Cr films: (**a**) 10 W, (**b**) 30 W, (**c**) 50 W, and (**d**) 70 W.

**Figure 3:**
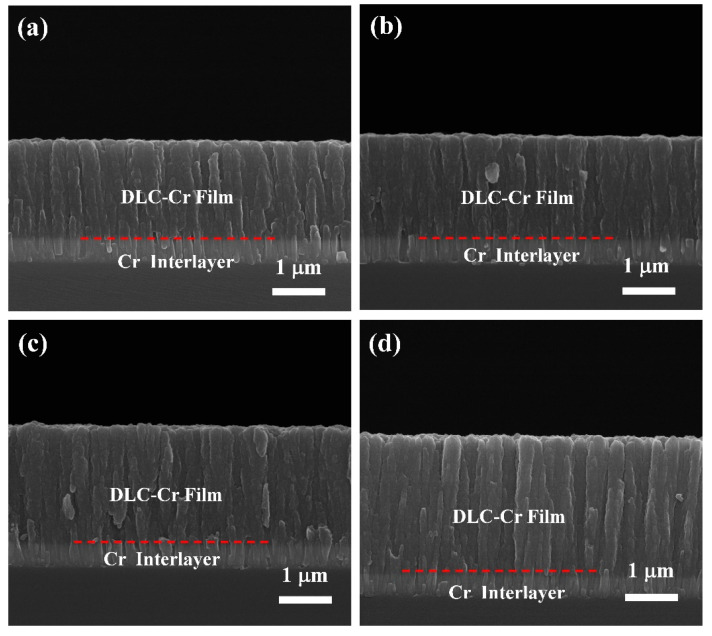
The cross-sectional morphologies of DLC-Cr films: (**a**) 10 W, (**b**) 30 W, (**c**) 50 W, and (**d**) 70 W.

**Figure 4:**
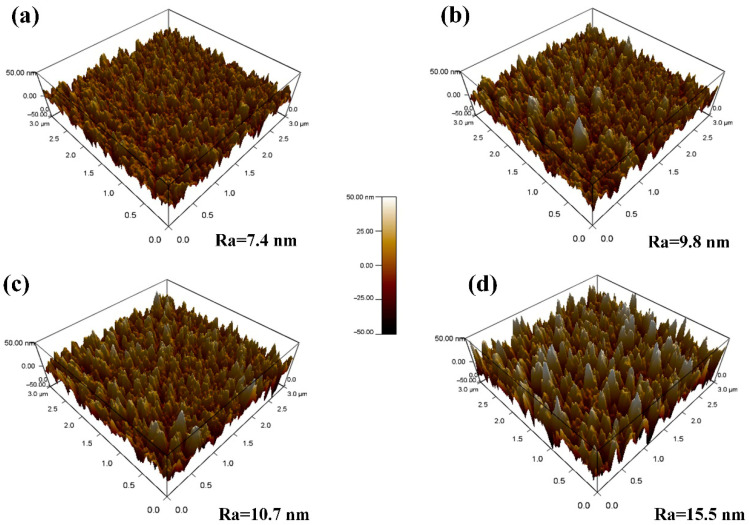
AFM three-dimensional surface topographies and corresponding RMS roughnesses of DLC-Cr films deposited at various Cr target powers: (**a**) 10 W, (**b**) 30 W, (**c**) 50 W, and (**d**) 70 W.

The surface morphology of the DLC-Cr films, as revealed by SEM in Figure 2, exhibits a significant dependence on the Cr target power (i.e., Cr content). At relatively lower powers, the films present a continuous and dense topography with a characteristic broccoli-like structure, comprising finely featured nodules. When the target power is increased to 50 W and 70 W, however, a distinct morphological transition occurs, and the surface evolves into a more pronounced granular structure with clear intergranular gaps. This evolution can be attributed to the improved Cr incorporation, which promotes the nucleation and growth of chromium carbide (CrC) nanocrystallites within the amorphous carbon matrix. The higher flux and energy of Cr species at elevated powers increase the adatom mobility, thereby facilitating surface diffusion and the coalescence of clusters into larger grains [19,33], ultimately leading to the formation of a nanocomposite structure. This microstructural reorganization, where the growing carbide phases define the granular features, is responsible for the observed surface roughening and the emergence of distinct interparticle boundaries. The presence of these nanocrystalline phases is further supported by the subsequent TEM and XPS analyses. Figure 3 presents the typical cross-sectional morphologies of the deposited films, which exhibit a well-defined bilayer structure consisting of a Cr interlayer and the DLC-Cr top layer. The Cr interlayer is deposited to enhance the adhesion between the film and substrate. It can be observed that all DLC-Cr layers developed a distinct columnar structure and extended through its entire thickness. With increasing Cr target power, a coarsening of the columnar structure within the DLC-Cr layer was observed, as shown in Figure 3a–d. This microstructural evolution is likely related to the enhanced Cr incorporation, which modifies the nucleation and growth kinetics, possibly via the formation of chromium carbide phases [20]. Concurrently, the overall thickness of film exhibits a gradual increase, a direct consequence of the enhanced deposition rate resulting from the higher sputtering yield at elevated target powers, as previously depicted in Figure 1. Similar surface and structural characteristics have been reported for other metal-doped DLC systems [2,34]. To further quantify the surface topography, AFM measurements were performed, and the three-dimensional morphologies along with the corresponding root mean square (RMS) roughness values are presented in Figure 4. The film surfaces are characterized by randomly distributed particle-like protrusions with varying sizes, and their roughness evolves from 7.4 nm to 15.5 nm as the target power increases from 10 W to 70 W. This trend is consistent with the 3D topographies (Figure 4a–d), which display a progressive coarsening of surface features and an enlargement of intergranular gaps. This roughening phenomenon is consistent with the SEM morphology shown in Figure 2. It is thought that the development of carbide nanocrystallites promotes the growth of larger surface protrusions, consequently leading to the increased roughness [33,35].

**Figure 5:**
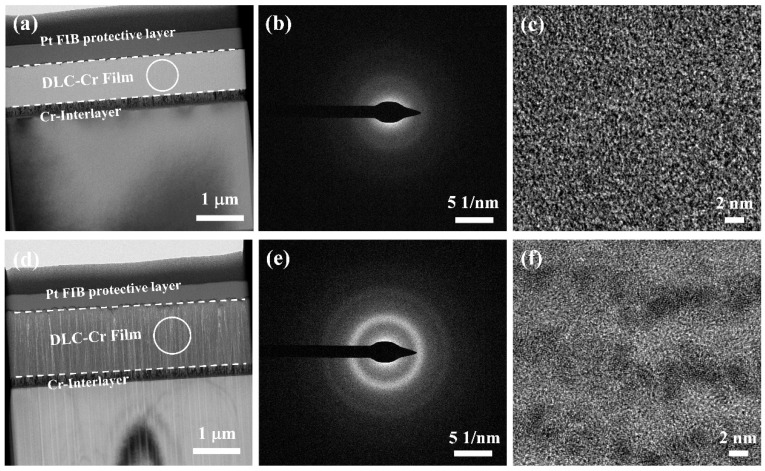
TEM analysis of DLC-Cr films: (**a**–**c**) cross-sectional TEM, SAED, and HRTEM images for 10 W film; (**d**–**f**) cross-sectional TEM, SAED, and HRTEM images for 30 W film.

To gain further insight into the microstructure evolution of the films, TEM analysis was conducted on the films deposited at 10 W and 30 W, as presented in Figure 5. For the film deposited at a low power of 10 W, the cross-sectional image (Figure 5a) clearly reveals a well-defined Cr interlayer and the overlying DLC-Cr layer. The corresponding selected-area electron diffraction (SAED) pattern (Figure 5b) exhibits only broad and diffuse halos, which is a characteristic of a fully amorphous structure [26]. This is further verified by the high-resolution TEM (HRTEM) image (Figure 5c), which shows a homogeneous contrast without any detectable crystal lattice fringes. This implies that the film had an amorphous structure at a low Cr target power. In contrast, the film deposited at 30 W displays a notable microstructural transformation. The cross-sectional view (Figure 5d) indicates an increased thickness and a more developed columnar morphology. The SAED pattern (Figure 5e) shows faint but distinct diffraction rings superimposed on the amorphous halo, suggesting the emergence of nanocrystalline phases. This is directly confirmed by the HRTEM image (Figure 5f), where discrete nanoparticles with darker contrast are observed, randomly distributed within the amorphous carbon matrix. This observed characteristic confirms the development of a nanocrystalline/amorphous composite, where chromium carbide nanocrystallites are incorporated into the amorphous carbon matrix.

**Figure 6:**
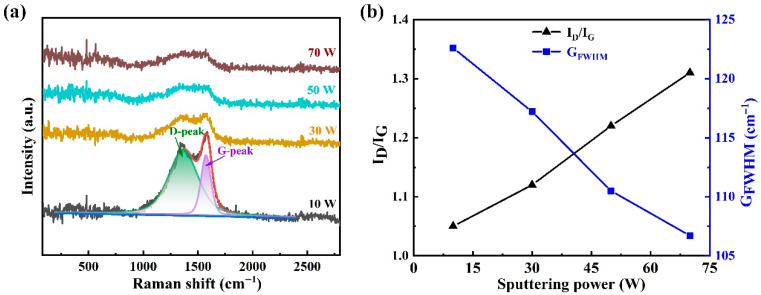
Raman spectra of DLC-Cr films deposited at different Cr target powers: (**a**) typical Raman curves, and (**b**) the variation in I_D_/I_G_ ratio and G_FWHM_ value with various Cr target powers.

Raman spectroscopy was employed to investigate the carbon bond structure of Cr-DLC films, and the results are summarized in Figure 6. All spectra (Figure 6a) exhibit a broad and asymmetric band between 1000 and 1800 cm^−1^, a typical characteristic of amorphous carbon. This broad band was fitted with two distinct peaks: the D band at ~1350 cm^−1^, which originates from the breathing mode of sp^2^ carbon in aromatic rings, and the G band at ~1580 cm^−1^, arising from the bond-stretching vibrations of sp^2^ carbon atoms in both rings and chains [28,36]. With increasing Cr target power, the overall intensity of both bands decreases, reflecting the reduced carbon content in the films. Usually, the fitted area ratio of the D to G bands (I_D_/I_G_) serves as a sensitive indicator of the structural order within the carbon network. As plotted in Figure 6b, the I_D_/I_G_ ratio shows a monotonic increase with higher target powers. This upward trend signifies an enlargement in the size and/or number of sp^2^-C carbon clusters and a corresponding increase in graphitic ordering [14,29]. In addition, the full width at half maximum of the G peak (G_FWHM_) was associated with graphite disorder. As shown in Figure 6b, the G_FWHM_ decreased with the increase in target power, indicating that the fraction of sp^3^-C carbon in the film is decreasing. The incorporation of Cr atoms is posited to catalyze this structural transformation by facilitating the conversion of sp^3^-C bonds to the more stable sp^2^ configuration and promoting the reorganization of the carbon network. This metal-induced graphitization effect, which lowers the overall stress, is a key factor underpinning the property trends observed in the mechanical and tribological tests.

**Figure 7:**
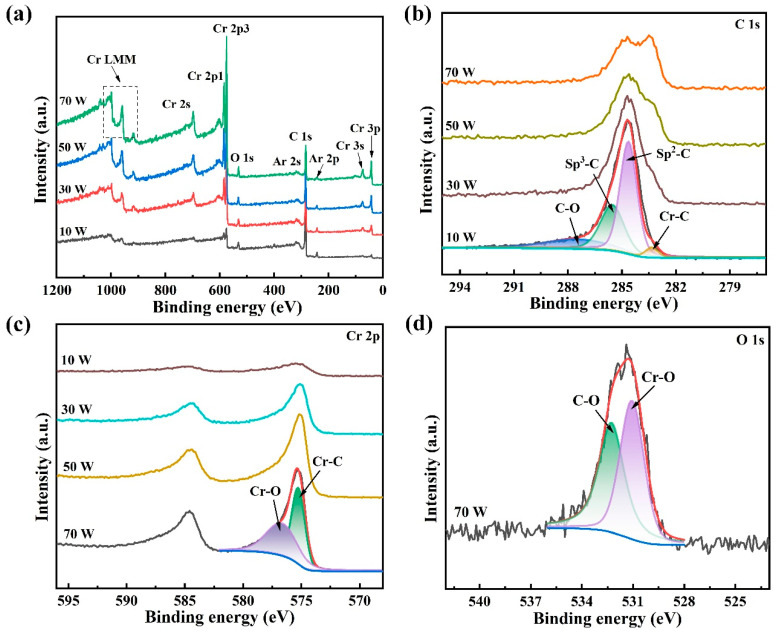
XPS results of DLC-Cr films: (**a**) survey spectra, (**b**) C 1s high-resolution spectra, (**c**) Cr 2p high-resolution spectra, and (**d**) O 1s high-resolution spectrum.

X-ray photoelectron spectroscopy (XPS) was conducted to investigate the evolution of atomic bonding states within the DLC-Cr films. The survey spectra in Figure 7a confirm the co-existence of C and Cr as the primary elements, successfully verifying the incorporation of Cr into the carbon matrix. A weak O signal is consistently observed, and is attributed to residual oxygen in the deposition chamber or post-deposition surface adsorption. In Figure 7b, the overall intensity of the C 1s spectra decreases monotonically with increasing target power, reflecting a gradual reduction in carbon content, which is consistent with the corresponding increase in Cr 2p signal intensity (Figure 7c). The deconvolution of the representative C 1s spectrum (10 W sample) showed four distinct peaks, with binding energies located at approximately the 287.5 eV, 285.5 eV, 284.6 eV, and a lower binding energy of 283.0 eV, corresponding to C-O, sp^3^-C, sp^2^-C, and the Cr-C bonds [20,37], respectively. The identification of this Cr-C component provides direct chemical evidence for the formation of chromium carbide, consistent with the nanocomposite structure observed in the TEM analysis. The formation of strong Cr-C bonds, as directly evidenced by XPS, preferentially stabilizes the sp^2^-hybridized configuration and facilitates the conversion of sp^3^-C sites to sp^2^-C sites. This further explains the increased I_D_/I_G_ ratio from the Raman result. The corresponding Cr 2p spectrum for the highly doped film (70 W) was fitted with two main peaks, as shown in Figure 7c: a dominant peak at around 574.8 eV assigned to the Cr-C bond and a weaker peak, approximately at 576.3 eV, corresponding to the Cr-O bond [38,39]. It is noteworthy that the binding energies for metallic Cr and chromium carbide are close, making the Cr 2p spectrum less definitive for their differentiation. Therefore, the presence of the low-energy component in the C 1s spectrum (~283.0 eV) serves as a more reliable indicator of carbide formation. Furthermore, it should be noted that the appearance of the Cr-O component in the Cr 2p spectra is mainly attributed to surface oxidation induced by post-deposition exposure to ambient air, which is a common phenomenon for Cr-containing films. The O 1s spectrum (Figure 7d) was deconvoluted into two components with binding energies centered at 531.0 eV and 532.2 eV, which were assigned to Cr-O and C-O bonds, respectively [20,39]. These results, combined with the TEM and XPS analyses, confirm that chromium primarily exists in the form of carbides within the films, corresponding to nanocrystalline phases dispersed in the amorphous carbon matrix.

**Figure 8:**
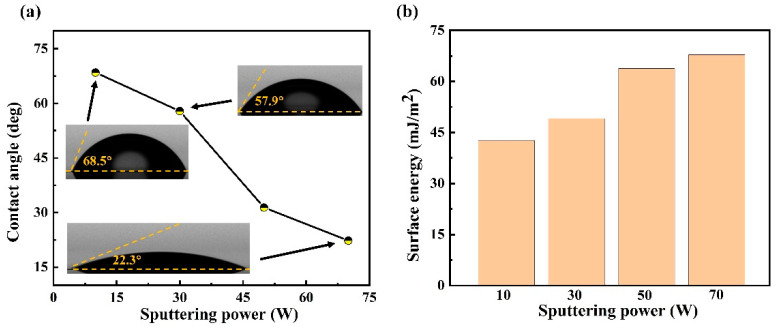
Water contact angle (**a**) and calculated surface energy (**b**) of DLC-Cr films deposited at various Cr target powers.

The wettability of the DLC-Cr films was evaluated by measuring the water contact angle, as shown in Figure 8a. All films exhibit contact angles below 90°, indicating hydrophilic behavior. With the Cr target power raised from 10 W to 70 W, the contact angle decreased significantly from 69.9° to 22.3°, while the corresponding surface energy (Figure 8b) increased from 41.77 mJ/m^2^ to 67.87 mJ/m^2^. This trend suggests that the Cr incorporation enhances the films’ affinity for water. According to the Wenzel’s model [40], the surface roughness can amplify the intrinsic wettability of a material, meaning that rougher hydrophilic surfaces tend to exhibit smaller contact angles. In the present study, the AFM results revealed an increase in surface roughness with an increase in Cr target power. Combined with the intrinsic hydrophilicity of the DLC-Cr films, this roughness increase can explain the observed reduction in contact angle. Furthermore, the increased surface energy is likely associated with the higher concentration of metallic Cr and the presence of Cr-O and Cr-C bonds, which introduce polar surface sites and thereby improve surface wettability [15,41]. These results suggest that both surface morphology and chemical composition play key roles in determining the wettability behavior of the DLC-Cr films.

**Figure 9:**
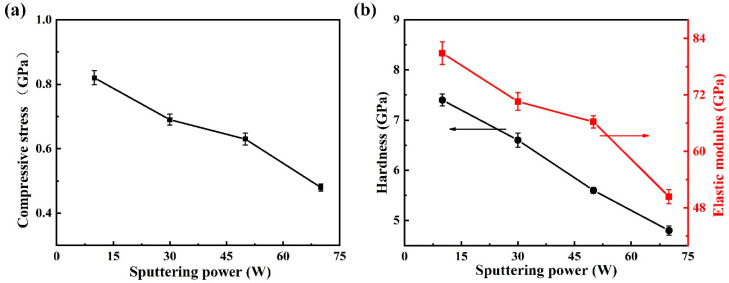
The compressive stress (**a**,**b**) hardness and elastic modulus of DLC-Cr films as a function of Cr target power.

To clarify the effect of Cr target power on the mechanical behavior of the films, the variations in residual stress, hardness (*H*), and elastic modulus (*E*) were analyzed, as illustrated in Figure 9. The residual stress values (Figure 9a) exhibit a clear decreasing trend from 0.82 at 10 W to 0.48 GPa at 70 W, indicating that the incorporation of Cr effectively relaxes the internal stress within the DLC matrix. This reduction in stress can be ascribed to two primary factors. First, the enhanced ion bombardment at higher sputtering powers increases adatom mobility on the growing surface, thereby promoting atomic rearrangement and facilitating stress relaxation during film growth [42]. Second, according to the Raman and XPS analyses, the increasing Cr content leads to a progressive reduction in the fraction of sp^3^-hybridized carbon and a corresponding enhancement in graphitic (sp^2^-C) ordering. Since the internal stress in DLC films is closely related to the density of sp^3^-C bonds [36], this structural transformation towards a more sp^2^-rich configuration effectively lowers the overall residual stress. In addition, the formation of CrC nanocrystallites within the amorphous carbon network can further relieve localized strain, contributing to stress relaxation. In Figure 9b, both the hardness and elastic modulus of films exhibit similar decreasing trends with increasing Cr target power. The observed decline in *H* and *E* can be directly correlated with the microstructural and bonding evolution discussed above. The hardness of DLC-based films is primarily governed by the fraction of sp^3^-C bonds and the continuity of the carbon network [2,36]. The increased I_D_/I_G_ ratio, together with the formation of carbide nanophases, disrupts the integrity of the amorphous carbon framework, leading to the reduction in hardness and modulus. Moreover, the decrease in residual stress also plays a promoting role in reducing hardness. Overall, the results indicated that appropriate Cr incorporation can effectively balance the stress relaxation and mechanical performance, providing a pathway to simultaneously achieve low stress and acceptable hardness in DLC-Cr nanocomposite films.

**Figure 10:**
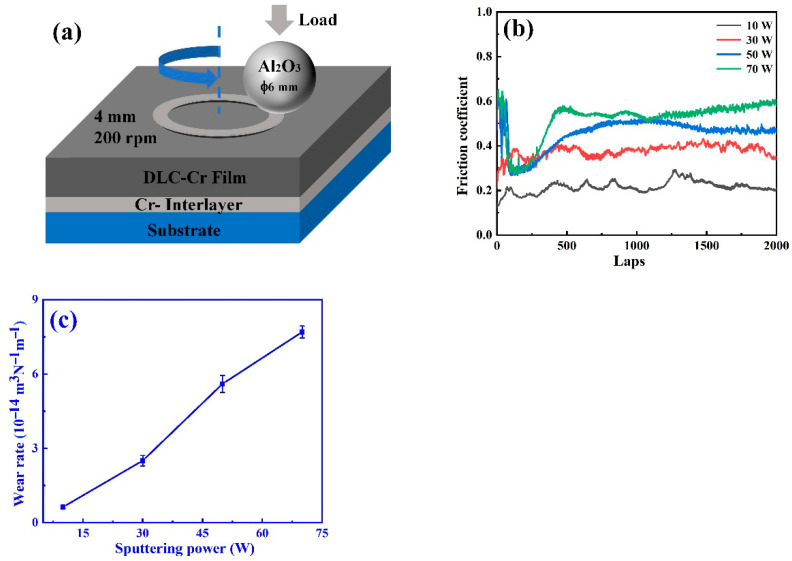
Schematic diagram of the ball-on-disk friction test (**a**); typical friction coefficient curves (**b**); and wear rate variation (**c**) of DLC-Cr films deposited under different Cr target powers.

The tribological behavior of the DLC-Cr films was investigated by using a ball-on-disk rotating friction tester, as schematically illustrated in Figure 10a. Figure 10b shows the friction coefficient (COF) evolution as a function of sliding laps for films deposited at various Cr target powers. All films exhibit two distinct stages: an initial running-in stage followed by a steady-state friction regime. The films prepared at lower Cr powers (10 W and 30 W) reach a stable friction stage rapidly and maintain relatively low COF values, whereas those deposited at higher powers (50 W and 70 W) display a slower transition and higher steady-state friction levels. The average COF increases from 0.21 at 10 W to 0.52 at 70 W, indicating that excessive Cr incorporation leads to a deterioration in lubricating performance [28]. Correspondingly, the calculated wear rate (Figure 10c) rises markedly from 0.63 × 10^−14^ m^3^/N·m to 7.7 × 10^−14^ m^3^/N·m with increasing target power, which consistent with the COF trend. The degradation in tribological performance with increasing Cr content can be attributed to several synergistic factors. Firstly, Raman and XPS analyses have revealed a progressive transformation of the carbon network to sp^2^-dominated graphitic and CrC-containing structures as Cr target power increases. The resulting decrease in the sp^3^-C fraction reduces the film’s hardness and load-bearing capability, thereby facilitating local deformation and microfracture during sliding. Secondly, the formation of dispersed carbide nanocrystallites interrupts the continuity of the carbon matrix, weakening its ability to accommodate shear stress and enhancing the likelihood of abrasive wear. Additionally, the reduced internal stress at higher Cr levels lowers the interfacial adhesion strength, further contributing to increased wear rates [38,43]. The combination of these effects results in higher friction and faster material removal under a sliding contact zone.

**Figure 11:**
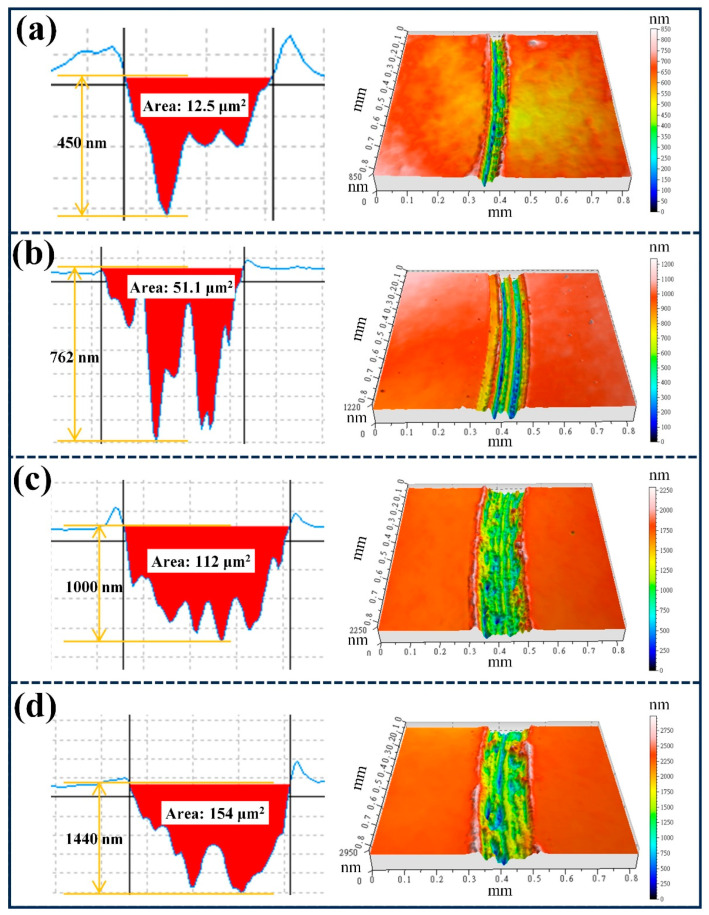
Three-dimensional (3D) wear track morphologies and two-dimensional (2D) cross-sectional profiles of DLC-Cr films after tribological testing at different Cr target powers: (**a**) 10 W, (**b**) 30 W, (**c**) 50 W, and (**d**) 70 W.

Figure 11 displays the three-dimensional wear track morphologies and corresponding two-dimensional cross-sectional profiles. The width and depth of the wear track increase significantly with Cr target power, which is consistent with the wear rate data. At low Cr powers, the wear tracks appear smooth and shallow, indicating mild adhesive wear with limited plastic deformation. In contrast, films deposited at higher powers (≥50 W) show broader and deeper wear scars accompanied by pronounced plowing grooves, which is characteristic of abrasive wear. These grooves are likely formed by hard CrC particles that detach from the film and act as third-body abrasives during sliding processes [44]. The transition from predominantly adhesive wear at low Cr contents to mixed abrasive-adhesive wear at high Cr contents reflects the underlying microstructural evolution and mechanical softening caused by excessive metal incorporation. Therefore, a moderate Cr content appears to provide the optimal balance between structural stability, hardness, and tribological performance, achieving low friction and superior wear resistance in DLC-Cr nanocomposite films.

## 4. Conclusions

Cr-doped DLC nanocomposite films were successfully synthesized using a hybrid HiPIMS/PDC magnetron sputtering system, in which the Cr content was effectively tailored by adjusting the Cr target power. With increasing target power, both the Cr content and deposition rate increased, accompanied by a gradual evolution of the film microstructure from an amorphous carbon matrix to a nanocomposite structure consisting of CrC nanocrystallites embedded in a graphitized carbon network.

The increased in sp^2^-C bond and carbide phase formation led to a reduction in residual stress, as well as distinct changes in surface morphology and roughness. Both the hardness and elastic modulus of films gradually decreased with increasing Cr target power, which was attributed to the diminished sp^3^-C fraction and the disruption of carbon network continuity. Although Cr incorporation facilitated graphitization, the concurrent mechanical softening and carbide particle generation resulted in a deterioration of tribological performance at higher Cr target powers, with friction coefficients increasing from 0.21 to 0.52 and wear rates rising accordingly. The wear mechanism evolved from mild adhesive wear to combined abrasive–adhesive wear. In addition, the wettability analysis showed that all films exhibited hydrophilic characteristics, with contact angles decreasing from 69.9° to 22.3° as Cr content increased, consistent with the rise in surface energy and roughness. Overall, moderate Cr incorporation achieved a favorable balance between structural stability, stress relaxation, and tribological behavior, providing a feasible strategy for tailoring the microstructure and multifunctional performance of DLC-based nanocomposite films.

## Data Availability

The data presented in this study are available on request from the corresponding author.

## References

[1] Jing P.P., Ma D.L., Gong Y.L., Luo X.Y., Zhang Y., Weng Y.J., Leng Y.X. (2021). Influence of Ag doping on the microstructure, mechanical properties, and adhesion stability of diamond-like carbon films. Surf. Coat. Technol..

[2] Ding J.C., Dai W., Zhang T.F., Zhao P., Yun J.M., Kim K.H., Wang Q.M. (2018). Microstructure and properties of Nb-doped diamond-like carbon films deposited by high power impulse magnetron sputtering. Thin Solid Films.

[3] Bae S.M., Horibata S., Miyauchi Y., Choi J. (2023). Tribochemical investigation of Cr- doped diamond-like carbon with a MoDTC-containing engine oil under boundary lubricated condition. Tribol. Int..

[4] Li H., Sun P., Lu Y., Zhang J., Liu B. (2025). The effects of Ti content on the wear and corrosion resistance of Ti-doped DLC films deposited on AZ91 surface via magnetron sputtering. Ceram. Int..

[5] Molak R., Topolski K., Spychalski M., Dulinska-Molak I., Moronczyk B., Pakieła Z., Nieużyła L., Mazurkiewicz M., Wojucki M., Gebeshuber A. (2019). Functional properties of the novel hybrid coatings combined of the oxide and DLC layer as a protective coating for AZ91E magnesium alloy. Surf. Coat. Technol..

[6] Peng Y., Peng J., Wang Z., Xiao Y., Qiu X. (2021). Diamond-like carbon coatings in the biomedical field: Properties, applications and future development. Coatings.

[7] Ferreira R., Martins J., Carvalho O., Sobral L., Carvalho S., Silva F. (2020). Tribological solutions for engine piston ring surfaces: An overview on the materials and manufacturing. Mater. Manuf. Process..

[8] Zhang W., Guo P., Yang Y., Li H., Yang W., Chen R., Xie B., Wang A. (2025). Carrier transport behavior and piezoresistive mechanism of Cr-DLC nanocomposite films. Appl. Surf. Sci..

[9] Lin Y., Zhou Z., Li K.Y. (2019). Improved wear resistance at high contact stresses of hydrogen-free diamond-like carbon coatings by carbon/carbon multilayer architecture. Appl. Suff. Sci..

[10] Shahsavari F., Ehteshamzadeh M., Amin M.H., Barlow A.J. (2020). A comparative study of surface morphology, mechanical and tribological properties of DLC films deposited on Cr and Ni nanolayers. Ceram. Int..

[11] Wang J., Tao X., Zhang X., Tian F., Huang Z., Tian W., Chen J. (2025). The influences from CrN transition layer thickness to the tribological and corrosion performance of DLC films. Diam. Relat. Mater..

[12] He Y., Su F., Sun J., Li Z., Liu Y. (2025). Microstructure and tribological properties of DLC films with varying Ti interlayer thicknesses on HNBR substrate deposited by PVD. Surf. Coat. Technol..

[13] Lai Z., Yang P., Zhang B., Zhu Y., Gao K. (2025). Effect of hydrogenation or not of DLC on its tribological properties in vapor and liquid methanol. Diam. Relat. Mater..

[14] Taiariol T.S., Martins G., Hurtado C., Tada D., Corat E., Airoldi V.T. (2025). Effect of individual and multiple incorporation of Ag and TiO_2_ on the properties of DLC films. Diam. Relat. Mater..

[15] Zhao D., Mei H., Ding J.C., Cheng Y., Zhang L., Zhang T.F., Kang H.K., Zheng J. (2023). Microstructure and properties of Mo doped DLC nanocomposite films deposited by a hybrid sputtering system. Vacuum.

[16] Ramirez V.H.T., Rodriguez G.C.M., Sanchez M.B., Olvera F.M., Diaz L.A.C., Martinez C.F., Carmona J.M.G. (2025). Effect of nitrogen doping on the mechanical and tribological properties of hydrogen-free DLC coatings deposited by arc-PVD at an industrial scale. Surf. Coat. Technol..

[17] Yu Z., Shang J., Wang Q., Zheng H., Mei H., Zhao D., Liu X., Ding J., Zheng J. (2025). Influence of Si content on the microstructure and properties of hydrogenated amorphous carbon films deposited by magnetron sputtering technique. Coatings.

[18] Zhang Y.G., Sun W.C., Dong Y.R., Ma M., Liu Y.W., Tian S.S., Xiao Y., Jia Y.P. (2021). Electrodeposition and microstructure of Ni and B co-doped diamond-like carbon (Ni/B-DLC) films. Surf. Coat. Technol..

[19] Zhang T.F., Wan Z.X., Ding J.C., Zhang S., Wang Q.M., Kim K.H. (2018). Microstructure and high-temperature tribological properties of Si-doped hydrogenated diamond-like carbon films. Appl. Suff. Sci..

[20] Mei H., Lu Z., Ding J.C., Liu S., Liu Y., Zhou M., Zheng J. (2025). Influence of Ar/C_2_H_2_ flow ratio on the microstructure and properties of Cr-containing DLC films deposited by medium frequency magnetron sputtering. J. Alloys Compd..

[21] Zhou Y., Wang X., Wang L. (2024). Microstructure and high-temperature tribological properties of Ti/Si co-doped diamond-like carbon films fabricated by twin-targets reactive HiPIMS. Diam. Relat. Mater..

[22] Evaristo M., Fernandes F., Cavaleiro A. (2023). Influence of the alloying elements on the tribological performance of DLC coatings in different sliding conditions. Wear.

[23] Su Y., Cai L., Huang W., Zhang T., Yu W., Zhang P., Hu R., Gong X. (2022). Improvement the tribological properties of diamond-like carbon film via Mo doping in diesel condition. Vacuum.

[24] Patnaik L., Maity S.R., Kumar S. (2020). Comprehensive structural, nanomechanical and tribological evaluation of silver doped DLC thin film coating with chromium interlayer (ag-DLC/Cr) for biomedical application. Ceram. Int..

[25] Balestra R.M., Castro A.M.G., Evaristo M., Escudeiro A., Mutafov P., Polcar T., Cavaleiro A. (2011). Carbon-based coatings doped by copper: Tribological and mechanical behavior in olive oil lubrication. Surf. Coat. Technol..

[26] Ding J.C., Mei H., Zheng J., Wang Q.M., Kang M.C., Zhang T.F., Kim K.H. (2021). Microstructure and wettability of novel Al containing diamond-like carbon films deposited by a hybrid sputtering system. J. Alloys Compd..

[27] Ding J.C., Chen M., Mei H., Jeong S., Zheng J., Yang Y., Wang Q., Kim K.H. (2022). Microstructure; mechanical, and wettability properties of Al-doped diamond-like films deposited using a hybrid deposition technique: Bias voltage effects. Diam. Relat. Mater..

[28] Zhou C., Xiao L.R.J., Zhang X., Mo X., Jiang A. (2025). Cr content regulates the friction and corrosion resistance of Cr/F-DLC thin films. Diam. Relat. Mater..

[29] Wang L., Wu Y., Yu S., Liu Y., Shi B., Hu E., Hei H. (2022). Investigation of hydrophobic and anti-corrosive behavior of Cr-DLC film on stainless steel bipolar plate. Diam. Relat. Mater..

[30] Sarakinos K., Alami J., Konstantinidis S. (2010). High power pulsed magnetron sputtering: A review on scientific and engineering state of the art. Surf. Coat. Technol..

[31] Shiri S., Ashtijoo P., Odeshi A., Yang Q.Q. (2016). Evaluation of Stoney equation for determining the internal stress of DLC thin films using an optical profiler. Surf. Coat. Technol..

[32] Rodriguez B.J., Schiller T.L., Proprentner D., Walker M., Low C.T.J., Shollock B., Sun H., Navabpour P. (2020). Effect of chromium doping on high temperature tribological properties of silicon-doped diamond-like carbon films. Tribol. Int..

[33] Hu Y., Wang S., Chai L., Wang P., Li Y. (2025). Effect of power on the microstructure and frictional properties of Si-DLC films prepared by high-power impulse magnetron sputtering (HiPIMS). Surf. Coat. Technol..

[34] Cao L., Liu J., Wan Y., Pu J. (2020). Corrosion and tribocorrosion behavior of W doped DLC coating in artificial seawater. Diam. Relat. Mater..

[35] Pang X., Shi L., Wang P., Xia Y., Liu W. (2009). Influence of methane flow on the microstructure and properties of TiAl-doped a-C:H films deposited by middle frequency reactive magnetron sputtering. Surf. Interface Anal..

[36] Robertson J. (2002). Diamond-like amorphous carbon. Mater. Sci. Eng. R Rep..

[37] Greczynski G., Primetzhofer D., Hultman L. (2018). Reference binding energies of transition metal carbides by core-level x-ray photoelectron spectroscopy free from Ar^+^ etching artefacts. Appl. Surf. Sci..

[38] Zheng J., Shang J., Zhuang W., Ding J.C., Mei H., Yang Y., Ran S. (2024). Structural and tribomechanical properties of Cr-DLC films deposited by reactive high power impulse magnetron sputtering. Vacuum.

[39] https://srdata.nist.gov/xps/EnergyTypeElement.

[40] Neumann A. (1974). Contact angles and their temperature dependence: Thermodynamic status, measurement, interpretation and application. Adv. Colloid Interf. Sci..

[41] Sun L.L., Guo P., Li X.W., Wang A.Y. (2017). Comparative study on structure and wetting properties of diamond-like carbon films by W and Cu doping. Diam. Relat. Mater..

[42] Santiago J.A., Martinez I.F., Lopez J.C.S., Rojas T.C., Wennberg A., Gonzalez V.B., Aldareguia J.M.M., Monclus M.A., Arrabal R.G. (2020). Tribomechanical properties of hard Cr-doped DLC coatings deposited by low-frequency HiPIMS. Surf. Coat. Technol..

[43] Guo C.Q., Li H.Q., Peng Y.L., Dai M.J., Lin S.S., Shi Q., Wei C.B. (2022). Residual stress and tribological behavior of hydrogen-free Al-DLC films prepared by HiPIMS under different bias voltages. Surf. Coat. Technol..

[44] Kim J.I., Lee W.Y., Tokoroyama T., Murashima M., Umehara N. (2023). Tribo-chemical wear of various 3d-transition metals against DLC: Influence of tribo-oxidation and low- shear transferred layer. Tribol. Int..

